# Endovascular treatment of acute limb ischemia for persistent sciatic artery aneurysms: a report of 2 cases

**DOI:** 10.1186/s42155-025-00568-5

**Published:** 2025-06-18

**Authors:** Eiji Koyama, Kazuki Tobita, Shun Sawada, Motoaki Kai, Hirokazu Mityashita, Shigeru Saito

**Affiliations:** Department of Cardiology, Shonan Kamakura General Hospital, 1370-1 Okamoto, Kanagawa, Kamakura, 247-8533 Japan

**Keywords:** Acute limb ischemia, Persistent sciatic artery, Endovascular therapy, Stent graft, Case report

## Abstract

**Background:**

Persistent sciatic artery (PSA) is a rare congenital anomaly associated with various complications, including atherosclerotic changes and aneurysms. These changes can cause limb ischemia, thrombosis, distal embolization of the PSA, rupture of aneurysms, buttock pain, and sciatica due to compression of adjacent tissues.

Acute limb ischemia (ALI) is a life-threatening condition. Treatment of ALI includes surgical and endovascular treatments (EVT); EVT includes catheter-directed thrombolysis (CDT) and angioplasty, with thrombolysis being highly effective. In Japan, urokinase is the only insurance-covered thrombolytic agent approved for ALI treatment; however, it is currently unavailable due to manufacturing issues.

**Case presentation:**

This case report details the treatment of two women (aged 89 and 82 years) with ALI associated with PSA. In both cases, reperfusion was achieved without CDT and stent grafts were deployed across the PSA aneurysm. The final angiogram showed that the PSA aneurysms had disappeared, and the vessel runoff was maintained. Both patients were successfully discharged from the hospital and experienced no complications over the next 6 months.

**Conclusions:**

Two patients with ALI with PSA were treated with EVT without CDT. These cases suggest that EVT without CDT may rescue ALI caused by PSA. Moreover, no standard treatment for sciatic artery remnants has been established. Endovascular treatment with stent grafts may be an option for older patients.

## Background

A persistent sciatic artery (PSA) is a rare congenital anomaly, with an incidence ranging from 0.01% to 0.06%. It is associated with various complications, including atherosclerotic changes or aneurysms that are often mechanically compressed due to their anatomical location. This can cause limb ischemia, thrombosis or distal embolization, aneurysm rupture, buttock pain, and sciatica due to the compression of adjacent nerves [1]. Acute limb ischemia (ALI) caused by PSA is uncommon because of its low prevalence [2]. Catheter-directed thrombolysis for PSA-presenting ALI has previously been shown to be effective [3]. However, urokinase, which is the only thrombolytic agent approved for ALI treatment by the Japanese insurance system, is currently unavailable owing to manufacturing issues.

We treated two cases of ALI with PSA using endovascular treatments (EVT) without a thrombolytic agent. Subsequently, the affected limbs were saved, and the patients recovered well, with no re-interventions required.

## Case presentation

### Case 1

An 89-year-old woman was admitted for subacute-onset claudication of the left leg that had developed over 3 days. Her height was 135.0 cm and body weight was 38 kg. Her medical history included hypertension and dyslipidemia; however, no arrhythmias were detected. She had undergone EVT with balloon angioplasty for occlusion of the popliteal artery due to a PSA aneurysm 3 years prior and was treated with edoxaban (30 mg p.o.).

**Fig. 1:**
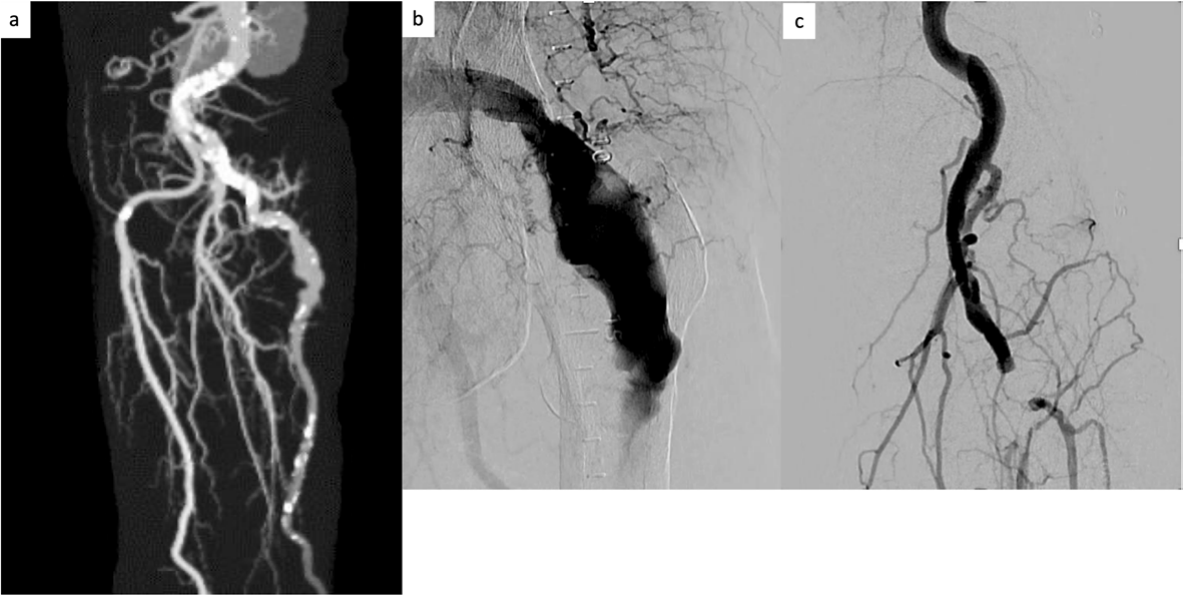
**a** Enhanced computed tomography angiography revealing a left PSA aneurysm and enhanced collateral flow toward the occluded PA below the knee joint, distal to a hypoplastic, incomplete SFA. **b**,** c** Angiography revealing aneurysmal dilatation of the proximal left PSA and total occlusion of the residual thrombus from the left distal PA to the tibioperoneal trunk. PSA, persistent sciatic artery; PA, popliteal artery; SFA

The resting ankle-brachial index in the left leg reduced to 0.77; duplex ultrasonography confirmed popliteal artery occlusion. The preoperative Society for Vascular Surgery (SVS) classification was Grade 2a [4]. Enhanced computed tomography revealed a left PSA aneurysm with thrombus and enhanced collateral flow towards the occluded popliteal artery (PA) below the knee joint, distal to a hypoplastic, incomplete SFA (Fig. 1a). She was diagnosed with PSA (Type 2b according to the Pillet–Gauffre classification) associated with lower-extremity artery disease, which required revascularization to improve her symptoms. Prasugrel was prescribed in addition to oral anticoagulants.

**Fig. 2:**
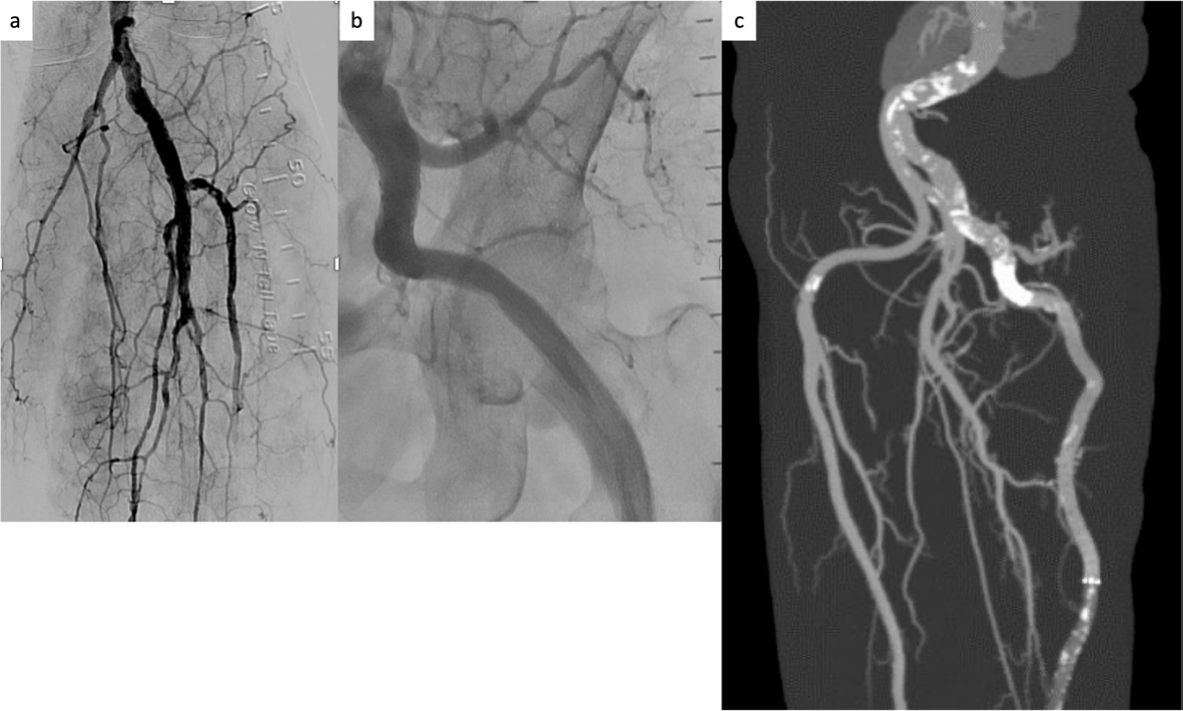
**a**,** b** Angiography revealing disappearance of the PSA aneurysms and two-vessel runoff. **c** Enhanced computed tomography angiography confirming disappearance of the PSA aneurysms. PSA, persistent sciatic artery

EVT was performed via the right common femoral artery using a 7-Fr Ansel (Cook Medical Japan, Tokyo, Japan). Digital subtraction angiography revealed aneurysmal dilatation of the proximal left PSA and filling of the defects in the left PA. Additionally, total occlusion was observed from the left distal PA to the tibioperoneal trunk (Fig. 1b, c). After completing multiple thrombus aspirations using an 8-Fr Thrombuster II (Kaneka Medics, Tokyo, Japan) as well as balloon inflation, blood flow to the anterior tibial artery was restored. Next, we examined the vascular lumen and vascular structure disruption using intravascular ultrasound. Subsequently, VIABAHN VBX (8.0 mm × 29 mm and 8.0 mm × 39 mm; W.L. Gore & Associates, Flagstaff, AZ, USA), and VIABAHN (8.0 mm × 250 mm; W.L. Gore & Associates) stent grafts were deployed across the PSA aneurysm. Post-dilatation was performed using a 12.0 mm × 20 mm semi-compliant balloon. The final angiogram showed that the PSA aneurysms had disappeared (Fig. 2a, b). The resting ankle-brachial index of the left leg improved to 1.12, and the patient was discharged from the hospital, with no complications reported over the next 3 years (Fig. 2c).

### Case 2

**Fig. 3:**
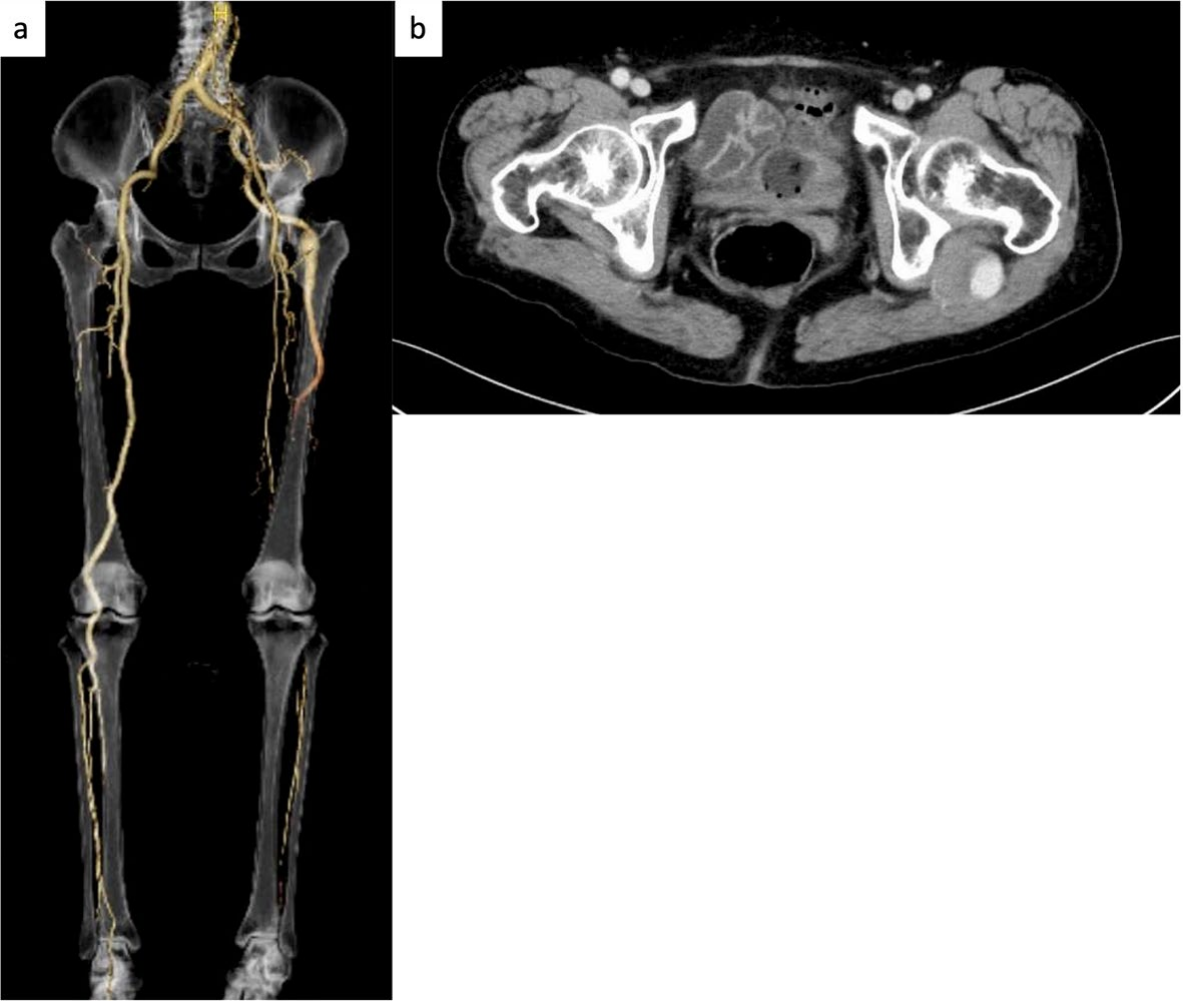
**a**,** b** Enhanced computed tomography angiography revealing a left PSA aneurysm and enhanced collateral flow toward the occluded PA below the knee joint distal to a hypoplastic, incomplete SFA. PSA, persistent sciatic artery; PA, popliteal artery; SFA

An 82-year-old woman was admitted with acute-onset left leg coldness and rest pain that had developed over 1 day. She had a history of hypertension, dyslipidemia, and type 2 diabetes mellitus, and was on oral therapy. Her height was 152 cm and body weight was 48 kg. Pulselessness in the popliteal and pedal arteries, pallor, and poikilothermia were observed in the left foot. Duplex ultrasonography revealed popliteal artery occlusion, with no signal below the knee artery. The resting ankle-brachial index could not be measured because of severe pain. Blood tests revealed elevated creatine kinase levels (4956 U/L; reference range 41–153 U/L). The preoperative SVS classification was Grade 2b [4]. Enhanced computed tomography revealed a left PSA aneurysm and enhanced collateral flow toward the occluded PA below the knee joint, distal to the hypoplastic, incomplete SFA (Fig. 3a, b). She was diagnosed with ALI associated with PSA (Type 2b according to the Pillet–Gauffre classification), which required emergency revascularization to salvage the limb.

**Fig. 4:**
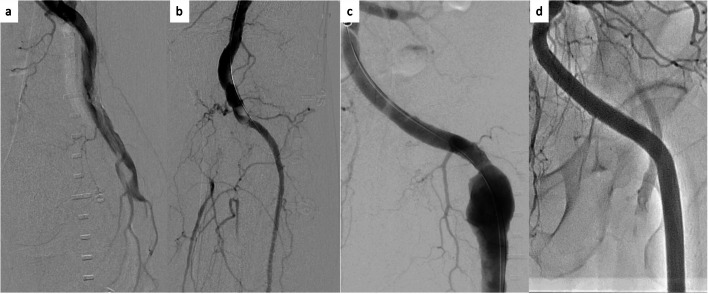
**a** Angiography revealing total occlusion of the residual thrombus from the left mid PA. **b** Angiography revealing disappearance of the PA embolization and restoration of blood flow in the anterior tibial artery. PA, popliteal artery. **c** Angiography revealing a left PSA aneurysm. **d** Angiography revealing disappearance of the PSA. PSA, persistent sciatic artery

Emergency EVT was performed via the right common femoral artery using a 6-Fr CROSSROADS (Nipro, Tokyo, Japan). Digital subtraction angiography revealed aneurysmal dilatation of the left internal iliac artery aneurysm, PSA, and filling defects in the left PA. The thrombus was occluded from the left PA to the tibioperoneal trunk. We performed multiple aspirations using an 8-Fr Mach1 (Boston Scientific, Marlborough, MA, USA), a GOGO catheter (Togo Medikit, Tokyo, Japan), and balloon inflation; blood flow to the anterior tibial artery was subsequently restored (Fig. 4a, b). As the procedure time exceeded 2 h and antithrombotic treatments were initiated on the same day, we chose to end the procedure by securing blood flow in the below-knee artery and treat the PSA aneurysm later. Prasugrel and apixaban were prescribed after the first EVT. Rehydration was continued after the procedure; the patient’s maximum creatine kinase level was 7300 U/L on the day after the first EVT.

Four days after the first EVT, a second EVT was performed to treat the PSA aneurysm via the right common femoral artery using an 8-Fr metal sheath (Teleflex, Tokyo, Japan). We examined the vascular lumen and structure disruption using intravascular ultrasound; subsequently, VIABAHN stent grafts (9.0 mm × 50 mm and 8.0 × 50 mm; W.L. Gore & Associates) were deployed across the internal iliac artery aneurysm. Post-dilatation was performed using an 8.0 mm × 100 mm semi-compliant balloon. The final angiogram showed that the stent grafts completely covered the internal iliac artery aneurysms, with no inflow into the aneurysms observed (Fig. 4c, d). The patient was discharged from the hospital and did not report any complications over the next 6 months.

## Conclusions

PSA can be treated surgically or endovascularly. Traditional surgical treatment for aneurysms, including bypass and resection, carries a high risk of injury to the sciatic nerve and has a narrow and deep operative field [5, 6]. Moreover, femoropopliteal bypass and aneurysm resection are highly invasive procedures that require general anesthesia. In contrast, EVT is associated with a lower risk of sciatic nerve injury and is less invasive than surgery. However, little is known regarding the long-term outcomes of EVT in terms of subsequent major amputations, development of ALI, and primary patency loss. Thus, attending physicians should consider anatomical factors as well as the patient’s background and condition when determining treatment strategies.

A recent systematic review showed that endovascular stent graft repair for PSA is safe in terms of periprocedural outcomes and may be a reasonable alternative to an open surgical approach [7]. Therefore, EVT can be considered the first choice for patients with PSA who require revascularization if they are not suitable for surgical treatment due to advanced age and comorbidities. EVT may also be opted for over surgery when treating complete PSA (Types 1, 2a, or 2b), which comprises 70–80% of all PSA cases. In these settings, surgical bypass treatment risks distal thromboembolization even after surgery is performed [1]. In our cases, we chose EVT after taking into consideration the presence of complete PSA and the advanced age of the patients.

ALI management must be considered when treating PSA. A systematic review of EVT for PSA found that 75% of cases presented as ALI, probably due to thromboembolism to the distal artery from the PSA aneurysm [7]. The treatment of ALI includes surgery and EVT; the latter includes catheter-directed thrombolysis and angioplasty, with thrombolysis being a highly effective treatment [8]. In Japan, only urokinase is covered by insurance for catheter-directed thrombolysis in peripheral arteries. However, urokinase is no longer produced due to manufacturing issues. One report demonstrated that EVT, including stent implantation, balloon angioplasty, percutaneous thrombus aspiration, or a combination of these techniques for ALI, can be performed without thrombolytic therapy [9]. Similarly, we successfully saved the affected limbs in our patients by performing EVT without the use of a thrombolytic agent. Therefore, EVT without thrombolysis might be effective even in cases of ALI due to PSA and could be considered when thrombolytic agents are not available. To the best of our knowledge, this is the first case report where treatment was completed without CDT. Although mechanical thrombectomy devices were not used in our cases, they can effectively remove large amounts of thrombi and may have an impact on clinical outcomes. Large-scale studies focusing on therapeutic options for ALI with PSA are warranted to establish standard care.

Two patients with ALI with PSA were treated with EVT without catheter-directed thrombolysis. Subsequently, the affected limb was saved in both patients, and the treatment course was good with no reintervention needed. Although no standard treatment has been established for sciatic artery remnants, endovascular treatment using stent grafts may be an option for older patients.

## Data Availability

Not applicable.

## Abbreviations

ALI: Acute limb ischemia
EVT: Endovascular treatment
CDT: Catheter-directed thrombolysis
PA: Popliteal artery
PSA: Persistent sciatic artery
